# Iron-Containing Oral Contraceptives and Their Effect on Hemoglobin and Biomarkers of Iron Status: A Narrative Review

**DOI:** 10.3390/nu13072340

**Published:** 2021-07-09

**Authors:** Jordie A. J. Fischer, Carolina S. Sasai, Crystal D. Karakochuk

**Affiliations:** 1 Food, Nutrition and Health, University of British Columbia, 2205 East Mall, Vancouver, BC V6T 1Z4, Canada; jordie.fischer@ubc.ca (J.A.J.F.); carolinasasai@alumni.ubc.ca (C.S.S.); 2 Healthy Starts, BC Children’s Hospital Research Institute, 938 West 28th Ave, Vancouver, BC V5Z 4H4, Canada

**Keywords:** iron, ferrous iron, ferritin, iron deficiency, iron-containing oral contraceptives, hemoglobin, anemia, oral contraceptive, contraceptive, birth control

## Abstract

Oral contraceptive use has been associated with decreased menstrual blood losses; thus, can independently reduce the risk of anemia and iron deficiency in women. Manufacturers have recently started to include supplemental iron in the non-hormonal placebo tablets of some contraceptives. The aims of this narrative review are: (i) to describe the relationship between oral contraceptive use and both anemia and iron status in women; (ii) to describe the current formulations of iron-containing oral contraceptives (ICOC) available on the market; and (iii) to systematically review the existing literature on the effect of ICOC on biomarkers of anemia and iron status in women. We discovered 21 brands of ICOC, most commonly including 25 mg elemental iron as ferrous fumarate, for seven days, per monthly tablet package. Our search identified one randomized trial evaluating the effectiveness of ICOC use compared to two non-ICOC on increasing hemoglobin (Hb) and iron status biomarker concentrations in women; whereafter 12 months of contraception use, there were no significant differences in Hb concentration nor markers of iron status between the groups. ICOC has the potential to be a cost-effective solution to address both family planning needs and iron deficiency anemia. Yet, more rigorous trials evaluating the effectiveness of ICOC on improving markers of anemia and iron deficiency, as well as investigating the safety of its consumption among iron-replete populations, are warranted.

## 1. Introduction

Contraceptives are an unparalleled innovation in the public health sector, allowing for family planning and pregnancy prevention [1], with estimates of 922 million users worldwide [2]. Contraceptives can be categorized into two methods: hormonal and non-hormonal contraception. The former includes contraception methods, such as hormonal intrauterine devices (IUD), oral contraceptive pills, and the vaginal ring [3]. The latter consists of barrier methods (male or female condom, with or without spermicide), copper IUD, the withdrawal method [3], and fertility awareness-based methods (FAM) [4], characterized by observing the physical signs that change throughout the different phases of a woman’s menstrual cycle along with hormone fluctuations to predict and monitor the fertile and infertile days. The importance of universal access to family planning education and services is underscored by the global commitment made in the Sustainable Development Goal target 3.7.1—that by 2030, access to sexual and reproductive healthcare services could be ensured to all, and that reproductive health be integrated into national strategies and programs [5].

Based on the data compiled for the report Contraceptive Use by Method 2019 by the United Nations, approximately 16% of women use oral contraceptives [2]. This report is based on data from 1247 surveys collected between 1950 and 2018 in 195 countries among women of reproductive age, defined as 15 to 49 years of age. Commonly known as the birth control pill or “the pill,” oral contraceptives are widely used. Reported rates of use among women are approximately 19% in Europe, 17% in Oceania, 15% in North America, 15% in Latin America, and 5% in Asia [2]. Oral contraceptives typically contain varying levels of both estrogen and progesterone (known as ‘combined contraceptives’), or solely progesterone [1,6]. With proper use, the effectiveness of oral contraceptives to prevent pregnancy can be up to 99%, though this is rarely achieved due to the challenge of remembering to consume it at the same time each day [6]. The physiological mechanism of oral contraceptive pills includes preventing the release of an egg from the ovaries, thickening the cervical mucus (making it harder for sperm to reach the uterus), and thinning the uterine lining to stop the uterus implantation of a fertilized egg [6].

Generally, oral contraceptives come in packets of 21 hormonal tablets followed by seven inactive placebo tablets. These placebo pills are void of any medical benefits and are given to mimic natural menses through a withdrawal bleed, while allowing women to continue the habit of consuming one tablet daily.

Beyond pregnancy prevention, women also use oral contraceptives for other reasons, as the synthetic hormones included in oral contraceptives have been shown to have other positive health benefits [7]. Some women consume oral contraceptives to manage acne, menstrual cramps, or heavy menstrual bleeding [6]. Heavy blood loss from menstruation is a potential contributor to iron deficiency anemia [8]. Defined as a blood loss of ~80 mL during one menstrual cycle [9], studies have shown that heavy menstrual bleeding affects ~18–38% of women of reproductive age [10]. However, the proportion of women thought to experience heavy menstrual bleeding is likely even higher due to underdiagnosis [10]. Prolonged heavy menstrual blood may lead to decreased iron stores in women, especially if women are not consuming a sufficient amount of dietary iron [10].

Oral contraceptives are known to reduce the duration and amount of menstrual blood loss throughout the menstrual cycle, potentially resulting in lower menstrual iron losses [11]. Interestingly, oral contraceptive users have been shown to have higher serum iron levels compared to non-oral contraceptive users [12,13,14,15]. In a population survey of 676 premenopausal Danish women (>35 years of age), serum ferritin concentrations were found to be inversely associated with the duration of menses (*p* < 0.0001) [15]. Further, in the same study, the duration of menses for women consuming oral contraceptives was shorter than those not consuming oral contraceptives, and median serum ferritin concentrations were ~62 μg/L vs. ~42 μg/L, respectively [15]. In another study of 268 healthy, menstruating, non-pregnant Danish women aged 18–30 years, serum ferritin concentrations were also observed to be inversely correlated with the duration of menstruation (spearman’s rank correlation coefficient (rs) = −0.25, *p* < 0.001) and perceived menstrual bleeding intensity (rs = −0.27, *p* < 0.001). Interestingly, some data suggest that oral contraceptive use may negatively impact a woman’s vitamin B_6_ status. Vitamin B_6_ is a cofactor in heme synthesis; however, a causal relationship between oral contraceptive use and anemia has not been established [16]. Further, women using oral contraceptives had a significantly shorter duration of menstruation than those using other methods of contraception, including IUDs [14]. In conclusion, there is strong evidence that oral contraceptives are associated with iron stores in women of reproductive age and have the potential to decrease monthly menstrual blood losses and directly impact the iron status of women.

Some manufacturers have recently started to include supplemental iron in place of the placebo tablets typically included (consumed during the week of the withdrawal bleed) with the goal of reducing the risk of anemia and iron deficiency among women. Iron is an essential mineral required for red blood cell production and oxygen transportation [17]. Iron deficiency is one of the most common nutritional deficiencies in the world [18]; it is characterized by low iron stores and is diagnosed by a ferritin concentration below a defined cut-off for a specific population [18]. Meeting dietary iron requirements is critical for women of reproductive age as they experience monthly iron losses during menstruation [10,19]. Iron deficiency anemia is common among women of reproductive age and can increase the risk of negative pregnancy outcomes [20] and impair work capacity [21]. There is strong evidence for the efficacy of iron supplementation to prevent and treat iron deficiency in both high and low-income countries [22]. In 2016, the World Health Organization issued a guideline recommending daily iron and folic acid (IFA) supplementation (30–60 mg elemental iron daily) for all menstruating adolescents and women for three consecutive months of the year in regions where anemia prevalence is ≥40% [23]. These recommendations are based on the presumption that iron deficiency causes ~50% of the global burden of anemia [23]. As a result of these international policies, iron supplements are widely distributed to women in many countries across the globe. To our knowledge, iron-containing oral contraceptives (ICOC) are currently available in North America [24,25,26,27,28,29,30,31,32,33,34], South Asia [35,36,37], West Asia [38], Southeast Asia [39,40,41], and Africa [42]. Yet, there is limited evidence to support the inclusion of this micronutrient in oral contraceptives; further, there are no data evaluating whether the inclusion of iron in ICOC is warranted or safe in iron-replete women.

The aims of this review are: (i) to describe the relationship between oral contraceptive use and both anemia and iron status in women; (ii) to describe the current forms of ICOC available on the market (including doses and countries of use); and (iii) to systematically review the existing literature on the effect of ICOC on biomarkers of anemia and iron status in women.

## 2. Materials and Methods

### 2.1. Search Methods

The following bibliographic online databases were searched for studies of ICOC to include in this review, from database initiation, up to and including 10 May 2020, following the population, intervention, comparator and outcomes (PICO) methodology [43]: Ovid MEDLINE, EMBASE, PubMed, Cumulative Index to Nursing and Allied Health Literature (CINAHL) and The Cochrane Central Register of Controlled Trials (CENTRAL) using the search terms “oral contracepti *”; “birth control”; “hormon *”; “pill”; AND “iron”; “ferrous”; “Fe”; “ferritin”; “h?emoglobin”, “iron deficien *”; and “an?emia” (Table S1). A grey literature search was undertaken on ClinicalTrials.gov, ProQuest Dissertations, and Thesis, manual searches of journals via Google Scholar, approved drug databases (including the World Health Organization (WHO)’s Prequalified Medicinal Products), and pharmaceutical websites using the same keywords. The search strategy was made in consultation with a librarian from the University of British Columbia. Reference lists of relevant articles were searched for more completed, ongoing, or unpublished studies or other relevant sources. Articles were restricted to those conducted in humans and published in English.

### 2.2. Selection Criteria and Eligibility

Primary research articles, consisting of intervention and observational trials, were included in this review. Eligible populations included women of reproductive age, aged 15–49 years, as per the World Health Organization definition. Articles were eligible if they included an oral contraceptive intervention group that included iron and a biochemical outcome marker of anemia or iron status, such as hemoglobin (Hb) and/or ferritin, soluble transferrin receptor (sTfR), serum iron (SI), total iron-binding capacity (TIBC), total body iron (TBI), or transferrin saturation (TS) concentration.

## 3. Results

### 3.1. Iron-Containing Oral Contraceptives

A summary list of the currently manufactured and distributed ICOC as reported in the searched literature is found in Table 1. This list may not be exhaustive, as some brands may be novel, while others may not be widely marketed or do not advertise or publish reports in English.

Of note, the first available ICOC is thought to be the now discontinued Con-fer, manufactured by Parke-Davis (Detroit, MI, USA) in 1974. Con-fer contained 21 hormonal tablets (1 mg norethisterone acetate and 0.05 mg ethinyl estradiol) and seven iron tablets containing 75 mg ferrous fumarate (~23.4 mg elemental iron/day, 164 mg/cycle) in each blister packet [44]. The stated advantage of this ICOC was that it ensured there are no recurring interruptions in therapy, as iron tablets are still taken on non-hormonal (placebo) days.

Seven companies have U.S. Food and Drug Administration (FDA) approved ICOC and market to the United States: AbbVie Inc. (Lake Bluffs, IL, USA) (formerly Allergan, Inc. (Dublin, Ireland)), Avion Pharmaceuticals (Alpharetta, GA, USA), Glenmark Pharmaceuticals (Mumbai, India), Mayne Pharma (Greenville, NC, USA), Pfizer (New York, NY, USA), Warner Chilcott (Dublin, Ireland), and Wyeth Pharmaceuticals (Madison, NJ, USA). Bangladesh, Cambodia, India, Israel, Nigeria, and the Philippines were also identified as the primary marketing populations of certain ICOC.

Limited data were found on the distribution and use of these ICOC, or if these brands are distributed to multiple countries beyond the country, they are manufactured in and/or primarily marketed to. For example, Levofem, which is produced in Indonesia, is imported and distributed by Deep K. Tyagi (DKT) International in Nigeria [40]. The same contraceptive is sold under the brand name “Lydia” in Ghana and “Lydia Rosa” in Myanmar [45], yet DKT still distributes both. DKT reports their contraception sales on their website, and in 2020, DKT and partner non-governmental organizations distributed >100 million oral contraceptives to >30 countries [46], with >90% of the contraceptives including iron (C. Purdy, DKT International, President, email communication 25 May 2021). More information is needed regarding the number of women consuming these ICOC and general consumption practices (e.g., duration of use). Further, no information could be obtained regarding whether these ICOC are purchased directly by women from health centres or facilities, or if they are procured by governments and/or non-governmental organizations and distributed via health centres and/or national programs.

Most brands of ICOC listed only the total amount of iron present in the tablet (not specifying the exact elemental iron dose, the total amount of iron available for absorption). For example, ferrous fumarate is 33% elemental iron by weight (75 mg iron, as ferrous fumarate, is equivalent to ~25 mg elemental iron). The daily dose of elemental iron in each tablet ranges from 10 to 25 mg, with the most common dose of ~25 mg elemental iron. One brand, Lo Loestrin^®^ Fe, provides only two days of supplemental iron per month, while other identified brands provide four or seven days of supplemental iron per month. All but one brand of ICOC contained iron in the form of ferrous fumarate (the exception being ferrous bisglycinate in Avion Pharmaceuticals’ ICOC (Balcoltra^®^, Alpharetta, GA, USA).

### 3.2. Trials Evaluating the Effect of Iron-Containing Oral Contraceptives on Biomarkers of Anemia and Iron Status

**Figure 1:**
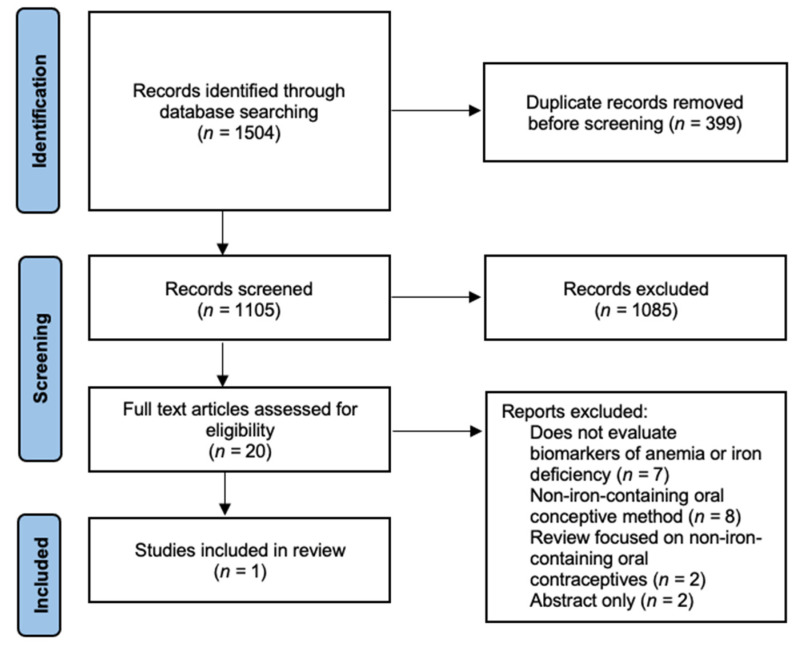
Flow diagram of search strategy.

Oral contraceptives have the potential to increase iron stores in their users by way of reducing menstrual iron losses, therefore decreasing iron deficiency [12,13,14,15]. As illustrated above, there are emerging brands of oral contraceptives, which include supplemental iron in the placebo tablets. Our aim was to examine the current body of evidence evaluating the use of ICOC and their effect on Hb and biomarkers of iron status. The Preferred Reporting Items for Systematic Reviews and Meta-Analyses (PRISMA) flow diagram presents the search strategy and flow for this review (Figure 1).

Our review identified only one randomized trial evaluating the effectiveness of ICOC use on increasing Hb and iron status biomarkers concentrations in women, conducted in Mexico in 1983 by Rivera et al. [47]. This study evaluated the effect of six different methods of contraceptive use: three types of oral contraceptives and three types of intrauterine devices. Hb, iron, and iron-binding capacity levels were measured at 6- and 12-month follow-up visits among 150 anemic women (Hb concentration 90–120 g/L), whose anemia was thought to be due to nutritional iron deficiency, not chronic disease, infection, or abnormal blood loss [47]. Women were randomized to one of six arms, including three oral contraceptive groups: oral contraceptive pills (0.15 mg levonorgestrel and 0.03 mg of ethynyl estradiol) for 21 days, followed by seven days of no-pill intake (Microgynon 21), the same oral contraceptive pills for 21 days, followed by seven days of placebo pills containing 75 mg of ferrous fumarate each (Microgynon 28 + iron group; ICOC), and the same oral contraceptive pills for 63 days followed by eight days of no-pill intake (Microgynon 63). In the ICOC group, there was a statistically significant increase in Hb concentrations observed after both 6 months, from mean ± standard deviation (SD), 10.53 ± 0.16 to 11.49 ± 0.32 g/dL (*p* < 0.01) and 12 months, 12.23 ± 0.38 g/dL (*p* < 0.001). There was also a significant increase in Hb concentration in the Microgynon 63 group at 6 months (mean ± SD, 10.93 ± 0.18 to 11.67 ± 0.33 g/dL; *p* = 0.025) and 12 months (mean ± SD, 11.68 ± 0.41 g/dL; *p* < 0.01). The increase in Hb was insignificant at 6 months for the Microgynon 21 group (*p* = 0.243) but was significant at month 12 (mean ± SD at baseline and 12 months: 10.83 ± 0.19 to 11.52 ± 0.34 g/dL; *p* = 0.031). All three oral contraceptive groups significantly increased serum iron levels at 6 months (*p* < 0.001) and 12 months following intervention (*p* < 0.001). There were no statistically significant differences between the ICOC and the two non-ICOC groups on Hb, serum iron and iron-binding capacity at 6- or 12-months [47]. Therefore, women in the ICOC group did not incur any benefit in Hb or serum iron increases over those in the non-ICOC groups. The study authors recommended that women with moderate anemia or low iron stores should consume ICOC, particularly in populations where the screening of anemia or iron status is not feasible, or the known prevalence of anemia or iron deficiency is high [47].

Additionally, in a multi-centre study across nine study centres in eight countries, researchers assessed the association of different forms of contraceptives including, combined oral contraceptives (non-iron-containing), depot injection (medroxyprogesterone acetate), subdermal implant (Norplant^®^), copper IUD, stainless steel ring IUD, and non-users of contraceptives on Hb and serum ferritin concentration in 2507 women (18–40 years) with anemia (Hb concentration < 120 g/L) over 12 months [48]. Oral contraceptive use was investigated in five study centres: Chengdu, Chiang Mai, Karachi, Santo Domingo, and Tunis. Hb concentration of oral contraceptive users was significantly greater in two study centre populations (*p* < 0.001, Chiang Mai; and *p* < 0.05, Santo Domingo) than non-contraceptive users. Serum ferritin concentration of oral contraceptive users was significantly greater in Chiang Mai (*p* < 0.001) than non-contraceptive users [48]. One of the exclusion criteria stated that women could not currently (or in the past three months) be taking any iron or other micronutrient supplements. Although this was screened for, after study completion and final analysis, it was found that women from five study centres were consuming ICOC (supplemental iron tablets with 75 mg ferrous fumarate, for seven of the 28-day contraceptive package). The final analyses were repeated, excluding the ICOC users; authors reported that the revised analyses were not notably different (no data reported) [48].

### 3.3. Attitudes towards Inclusion of Iron in Oral Contraceptives

Only one study was identified that evaluated the attitudes of both users and physicians concerning the inclusion of supplemental iron in oral contraceptives. The acceptance of ICOC was measured in a survey led by Richards-Brandt in 1988 in the United States [49]. The survey was administered to 192 obstetrician-gynecologists and 469 women oral contraceptive users to assess their attitudes towards a 28-day contraceptive pill regime where seven of the tablets contained iron rather than sugar placebos. This altered supplementation regime was designed to encourage user compliance as authors stated the poor acceptance of sugar tablets. Over 70% of health professionals and over 80% of women surveyed approved the use of ICOC, stating it to be a convenient method for remembering to stay on track with taking their daily oral contraceptives [49]. However, in this survey the authors did not investigate whether or not ICOC was an effective method to improve anemia and/or iron status.

## 4. Discussion

In certain populations and/or settings, ICOC has the potential to be a cost-effective alternative or addition to large-scale untargeted iron supplementation programs among women. As women of reproductive age are at risk for iron deficiency anemia [18,50], combining birth control and iron supplementation together can address both issues of family planning and iron deficiency anemia, with one cost-effective solution. Gebremedhin et al., in their paper on contraceptive use and Hb concentration in sub-Saharan Africa, also noted the potential of ICOC to contribute to anemia reduction [51]. In addition, acceptance rates for ICOC would likely be high, as women are already used to taking their daily pill and would not need to change their routines. Oral contraceptives have also been used as a vehicle to deliver other micronutrients, including folic acid, to increase blood folate concentrations in women of reproductive age to reduce the risk of neural tube defects [52].

On the contrary, there could also be potential harms of untargeted population-wide iron supplementation through the provision of ICOC; therefore, both the benefits and harms must be evaluated. The tolerable upper intake level (UL) (the highest average daily micronutrient intake level unlikely to increase risk of adverse health effects to most individuals in a certain population group) for iron is 45 mg/day for non-pregnant women aged 19–50 years, on account of the commonly experienced side effects of iron supplementation, such as gastrointestinal discomfort [53]. Although the common dose of elemental iron found in the ICOC was 25 mg, not exceeding the UL, it still has the potential to cause gastrointestinal discomfort, and at worst, iron overload in some at-risk individuals (e.g., those with severe genetic hemoglobin disorders). In iron-replete individuals, the consumption of high-dose oral iron may be harmful. This is because iron is a catalyst for oxidative and inflammatory reactions. Consuming excess iron can result in free iron, called non-transferrin-bound iron, which can increase reactive oxygen species production, leading to oxidative stress [54,55] and DNA and cellular damage [56,57,58]. Excess iron has been associated with diabetes and neuropathy [59,60]; while decreased growth [61], impaired development [62,63], and increased morbidity have been observed in infants and children [64]. In malaria-endemic regions, iron supplementation is known to increase the risk of infection [65]. Excess iron that is not absorbed in the duodenum passes into the colon, where it has been demonstrated to increase the susceptibility to pathogen growth and intestinal inflammation in child and infant populations [66,67,68]. The risk of adverse effects of iron supplementation appears to be highest among populations with a high enteric burden and those with poor water, sanitation, and hygiene standards [69].

Lastly, the opportunity for iron supplementation to cause injury may pose a greater risk to populations with certain genetic hemoglobin disorders, which are autosomal inherited conditions, such as sickle cell disease or hemoglobin E disorder, common in many areas of the world [70]. In the case of severe forms of these genetic hemoglobin disorders, iron metabolism may be altered, putting women at higher risk of iron overload [70]. Therefore, not all women may benefit from ICOC, even if the population has high prevalence rates of anemia.

Of note, in all brands of ICOC that we discovered, the daily dose of elemental iron did not exceed 25 mg, and the iron-containing supplements in the ICOC are only consumed up to a total of seven days each month; thus, the risk of consuming ICOC still appear relatively low, even for those women that are iron-replete or have genetic hemoglobin disorders. As red blood cells have a ~120 lifespan, it is unknown if seven days of 25 mg elemental iron would be sufficient to improve depleted iron stores in women with severe iron deficiency anemia [17]. However, in some populations or regions, women may be consuming supplemental iron from other iron interventions (such as iron and folic acid, in line with the WHO recommendations), fortified food products, and ICOC. Under these circumstances, women may be at risk as they are consuming supplemental iron from multiple sources.

The most common form of iron included in the identified ICOC was ferrous fumarate. Iron salts, including ferrous fumarate, are poorly absorbed [71]. Certain dietary components affect the bioavailability of iron by binding it in the gastrointestinal tract, inhibiting its absorption. Staple foods in low-resource settings are often low in highly bioavailable heme iron (e.g., meat) and high in iron inhibitors (e.g., phytates found in cereals, legumes, and leafy greens) [17]. Chelated iron, such as ferrous bisglycinate (used in ICOC Balcoltra^®^), has been shown to have greater bioavailability in oral supplementation and food fortification than conventional iron salts and results in less gastrointestinal side effects [72,73]. Future research assessing the inclusion of iron in oral contraceptives should query the most appropriate form of iron for inclusion.

Lastly, ICOC are not currently included in the WHO Model Essential Medicines List (MEML). This list is used to guide the selection of medicines, including nutritional supplements, for the primary health care needs of the population [74]. If a formulation is included on this list, it suggests that it should be available (or approved for procurement), in adequate amounts, at the appropriate dosage and price, within a working health care system [75]. While ICOC are not included on the WHO MEML, oral contraceptives (30 μg ethinyl estradiol + 150 μg levonorgestrel; 35 μg ethinyl estradiol + 1 mg norethisterone; 30 μg levonorgestrel; and 30 μg ulipristal (as acetate)), iron and folic acid (equivalent to 60 mg iron + 400 micrograms folic acid) and iron-only (equivalent to 60 mg iron) tablets are listed. If future research shows ICOC to be an effective method to prevent and/or treat iron deficiency and/or anemia, it may be important to include ICOC in the WHO MEML to ensure ICOC are approved for procurement and have adequate supply.

## 5. Conclusions

In our review, we discovered 21 different brands of ICOC that are currently being manufactured and marketed globally by 12 pharmaceutical companies. The daily dose of elemental iron in ICOC ranged from ~10–25 mg, with the most common dose being 25 mg. Very little research has been conducted to evaluate the effects of ICOC on biomarkers of anemia and iron status. Only one trial was identified in our search that compared the effects of ICOC to non-ICOC on Hb concentration and iron status in anemic women. There were no differences in Hb or serum iron concentrations among women receiving the ICOC and non-ICOC following 12 months of intervention. Therefore, no additional benefits of ICOC were observed; however, of note, adverse side effects were not assessed [47]. Despite this lack of evidence, numerous pharmaceutical companies continue to manufacture and distribute ICOC globally.

In some populations and/or settings, ICOC have the potential to be a cost-effective solution for the prevention and/or treatment of iron deficiency, which is one of the most common nutritional deficiencies globally [18]. Given that 16% of contraceptive users adopt the pill method, and menstruating women are known to be at risk of iron deficiency [2], women could potentially benefit from the inclusion of iron in oral contraceptives as a novel approach to address iron deficiency, as an alternative to iron or IFA supplementation or fortification programs. Still, research is warranted to assess the effectiveness of ICOC to increase Hb concentration and iron status, with the goal of preventing and/or treating iron deficiency. Considering the global widespread use of oral contraceptives, a rigorous elevation of both the benefits and harms of ICOC is needed, particularly in women who are iron-replete or have severe genetic hemoglobin disorders.

## Figures and Tables

**Table 1:** Summary list of current commercially available oral contraceptives containing iron.

Brand Name	Pharmaceutical Company	Reported Country of Use	Marketed Iron Dose (mg)	Elemental Iron Dose (mg)	Number of Iron Tablet Days/Total Number of Tablets in Monthly Package	Form of Iron	Relevant Information Found in Package Insert Regarding the Inclusion of Iron
Lo Loestrin^®^ Fe [24] Lo Minastrin™ Fe [25]	AbbVie Inc.	United States	75	~25	2/28	Ferrous Fumarate	The ferrous fumarate tablets do not serve any therapeutic purpose.
Taytulla™ [27] ESTROSTEP Fe™ [28]	AbbVie Inc.	United States	75	~25	4/28	Ferrous Fumarate	(Ferrous fumarate) does not serve any therapeutic purpose.
Balcoltra^®^ [29]	Avion Pharmaceuticals	United States	36.5	10	7/28	Ferrous Bisglycinate	The ferrous bisglycinate tablets do not serve any therapeutic purpose.
Levofem^®^ [42]	DKT International Nigeria	Nigeria	75	~25	7/28	Ferrous Fumarate	Contains ferrous fumarate for blood forming or repair ingredient by stimulating the formation of red blood cells.
Charlize^®^ [39] Trust Pill^®^ [40]	DKT Philippines Inc.	Philippines	75	24.75	7/28	Ferrous Fumarate	Ferrous fumarate is an iron supplement that helps improve the hemoglobin content of blood during menstruation.
Suvida^®^ [35]	Eskag Pharma	India	60	~25	7/28	Ferrous Fumarate	None listed.
Hailey™ Fe [30]	Glenmark Pharmaceuticals	United States	75	~25	7/28	Ferrous Fumarate	The ferrous fumarate tablets are present to facilitate ease of drug administration via a 28-day regimen, are non-hormonal, and do not serve any therapeutic purpose.
Lyta-28^®^ [37]	Incepta Pharmaceuticals	Bangladesh	75	~25	7/28	Ferrous Fumarate	None listed.
Microgestin^®^ Fe [31]	Mayne Pharma	United States	75	~25	7/28	Ferrous Fumarate	The ferrous fumarate tablets are present to facilitate ease of drug administration via a 28-day regimen, are non-hormonal, and do not serve any therapeutic purpose.
Zinnia-F [41]	Mylan Laboratories Limited	Cambodia	75	~25	7/28	Ferrous Fumarate	The other tablets (7 brown tablets) are hormone-free.
Norminest^®^ Fe [32] Norquest^®^ Fe [32]	Pfizer	United States	75	~25	7/28	Ferrous Fumarate	The iron tablets are not included for any therapeutic purpose but to provide a daily tablet regimen for days 22 through 28 of the cycle.
Junel^®^ 1/20 [38]	Teva Generics	Israel	75	~25	7/28	Ferrous Fumarate	The ferrous fumarate tablets are present to facilitate ease of drug administration via a 28-day regimen, are non-hormonal, and do not serve any therapeutic purpose.
Loestrin^®^ 24 Fe [33]	Warner Chilcott Company	United States	75	~25	7/28	Ferrous Fumarate	The ferrous fumarate tablets are present to facilitate ease of drug administration via a 28-day regimen, are non-hormonal, and do not serve any therapeutic purpose.
Minastrin™ Fe [34]	Warner Chilcott Company	United States	75	~25	4/24	Ferrous Fumarate	The ferrous fumarate capsules do not serve any therapeutic purpose.
Lo/Ovral^®^-28 [26] Ferrous Fumarate [26]	Wyeth Pharmaceuticals	United States	75	~25	7/28	Ferrous Fumarate	The hormone-free tablets containing ferrous fumarate should not be used in women with iron storage disorders, such as hemochromatosis and hemosiderosis.
Mala-N [36]	None reported	India	75	~25	7/28	Ferrous Fumarate	None listed.

DKT, Deep K. Tyagi.

## Supplementary Materials

The following are available online at https://www.mdpi.com/article/10.3390/nu13072340/s1, Table S1: Search strategy of Ovid MEDLINE(R) and Epub Ahead of Print, In-Process & Other Non-Indexed Citations and Daily 1946 to 10 May 2021.
