# Supplementary material for: Iron-Containing Oral Contraceptives and Their Effect on Hemoglobin and Biomarkers of Iron Status: A Narrative Review
Source: Nutrients. 2021 Jul 9;13(7):2340. doi: 10.3390/nu13072340 (PMC8308850; doi:10.3390/nu13072340)
Supplement: Supplementary file 1 [file nutrients-13-02340-s001.zip › nutrients-1273192-supplementary.pdf]

Table S1. Search strategy of Ovid MEDLINE(R) and Epub Ahead of Print, In-Process & Other Non-Indexed Citations and Daily 1946 to May 10, 2021.

#	Searches	Results
1	Contraceptives, Oral/	19341
2	((birth control or hormon* or contracepti*) adj2 (pill* or oral)).tw,kf.	30423
3	1 or 2	36950
4	Anemia, Iron-Deficiency/	10437
5	Iron Compounds/	3193
6	(iron or ferrous or ferritin or FE or h?emoglobin or an?emia).tw,kf.	543362
7	4 or 5 or 6	544721
8	3 and 7	765
9	limit 8 to (english language and humans)	514
